# The Role of Genetics in Risk Stratification Strategy of Dilated Cardiomyopathy

**DOI:** 10.31083/j.rcm2309305

**Published:** 2022-09-09

**Authors:** Anastasia Xintarakou, Ourania Kariki, Ioannis Doundoulakis, Petros Arsenos, Stergios Soulaidopoulos, Aggeliki Laina, Panagiotis Xydis, Athanasios Kordalis, Nikolaos Nakas, Alexia Theofilou, Charalampos Vlachopoulos, Konstantinos Tsioufis, Konstantinos A Gatzoulis

**Affiliations:** ^1^First Cardiology Department, National and Kapodistrian University of Athens, Hippokration General Hospital, 11527 Athens, Greece; ^2^Department of Cardiology, Onassis Cardiac Surgery Center, Athens, 17674 Kallithea, Greece; ^3^Department of Cardiology, General Hospital of Nikaia-Piraeus “Agios Panteleimon”, Piraeus, 18454 Nikaia, Greece

**Keywords:** dilated cardiomyopathy, risk stratification, genetic testing, prevention, sudden cardiac death

## Abstract

Dilated cardiomyopathy (DCM) is a heart disorder of diverse etiologies that affects millions of people worldwide, associated with increased mortality rate and high risk of sudden cardiac death. Patients with DCM are characterized by a wide range of clinical and pre-clinical phenotypes which are related with different outcomes. Dominant studies have failed to demonstrate the value of the left ventricular ejection fraction as the only indicator for patients’ assessment and arrhythmic events prediction, thus making sudden cardiac death (SCD) risk stratification strategy improvement, more crucial than ever. The multifactorial two-step approach, examining non-invasive and invasive risk factors, represents an alternative process that enhances the accurate diagnosis and the individualization of patients’ management. The role of genetic testing, regarding diagnosis and decision making, is of great importance, as pathogenic variants have been detected in several patients either they had a disease relative family history or not. At the same time there are specific genes mutations that have been associated with the prognosis of the disease. The aim of this review is to summarize the latest data regarding the genetic substrate of DCM and the value of genetic testing in patients’ assessment and arrhythmic risk evaluation. Undoubtedly, the appropriate application of genetic testing and the thoughtful analysis of the results will contribute to the identification of patients who will receive major benefit from an implantable defibrillator as preventive treatment of SCD.

## 1. Introduction 

Dilated cardiomyopathy (DCM) is a primary heart disorder with a prevalence of 1:250 individuals that can occur at any age but most frequently is presented during the young adulthood [1]. A diverse range of clinical phenotypes are included in the wider disease spectrum, beginning with asymptomatic individuals (with or without a disease associated genetic substrate) or patients with mild dilation, and resulting to significant hypokinesia whether or not accompanied by left ventricular enlargement [2]. DCM is considered a major health problem with meaningful social impact due to the adverse events of heart failure and sudden cardiac death (SCD) developing during the disease progression [3]. Although newer medical treatment have significantly improved the clinical manifestation of heart failure by ameliorating symptoms and delaying decompensation, SCD remains a dominant cause of mortality as it often affects young individuals, in an out of hospital setting, with low resuscitation rates [4].

Long-term research has revealed a diverse disease etiology including reversible and non-reversible causes, associated with acquired or genetic factors [5, 6]. From the late 20th century, when the development of next-generation sequencing became available beyond research purposes, until today, the number of DCM causative genes has been gradually increased, thus reclassifying several patients who were considered to have idiopathic disease [7, 8]. According to the latest data, approximately 100 genes have been detected as DCM related, while 19 of them represent sufficient evidence based on the Clinical Genome Resource (ClinGen) classification to be characterized as of “Definite”, “Strong” or “Moderate” disease relationship [9]. Both the American Heart Association and the European Society of Cardiology recognize the dual origin of DCM, proposing its classification either as a mixed cardiomyopathy or as a disorder of genetic or non-genetic etiology, respectively [6, 10]. Therefore, the final phenotypic expression could potentially be the result of a single cause or an amalgam of the overlapping action of many different factors [11].

Given the complexity of the disease, a comprehensive patients evaluation considering multiple parameters such as genetic, imaging, laboratory and clinical could probably provide the most accurate perception regarding the development of DCM and the potential outcome in each individual [12]. According to current guidelines, the prevention of SCD through an implantable cardioverter-defibrillator (ICD) is recommended either for individuals with reduced left ventricular ejection fraction (LVEF) (≤35%) and symptomatic heart failure or for DCM patients with a detected disease-causing mutations in lamin A/C (*LMNA*) gene and additional risk factors. However, the involvement of other parameters in treatment decision making is not yet clearly defined [13, 14].

The scope of the present study is to provide a thorough overview of the current knowledge about the genetic substrate associated with DCM development, as well as, to illuminate the contribution of a multifactorial process as part of the general spectrum of precision medicine for patients’ assessment and management. Finally, a discussion will be conducted regarding the current role and future perspectives of genetic testing in personalized patients’ evaluation.

## 2. Genetic Testing in DCM

The yield of identified pathogenic variants in patients with DCM is approximately 35% with the proportion to reach the 40% in familial disease and to be estimated less than 20% in sporadic cases [15]. By definition, a familial distribution of DCM is diagnosed when more than one member of the same family (first- or second-degree relatives) are affected by the disease and/or a death of unexplained cause at a young age has been recorded. At present, a familial base is detected in a rate between 20% and 30% of DCM patients, while according to two studies that conducted in the 80s, familial cardiomyopathy recorded only in 2% and 6% of the studied population [16, 17, 18, 19]. This difference in comparison to the past could be explained by both the widespread availability of clinical echocardiography or other imaging techniques and apparently the rapid progress in the field of genetics [11].

### 2.1 Types and Interpretation of Genetic Mutations

Genetic mutations are specific alteration of the deoxyribonucleic acid (DNA) nucleotides that affect the ribonucleic acid (RNA) sequence and as a consequence the resulting protein. Approximately 6 billion base pairs constitute the human genome in each single diploid cell (not gametes) and only 1% are classified as exons, that is, regions that encode proteins [20]. More than 3 million single-nucleotide polymorphisms (SNPs) can be detected in each individual when compared to the current reference human genome, however only the minority has a significant clinical impact leading to inherited diseases. Due to this complexity and diversity of the genetic substrate, determining the underlying meaning of each SNP and extracting only the disease-associated variants, is an undeniably challenging process that gradually upgraded as the sequencing of a larger proportion of the population is achieved [20, 21].

Most SNPs are observed in non-coding areas, without affecting protein synthesis or termed “synonymous” if they not result in amino acid modification even though they are located in regions of coding DNA. On the contrary, non-synonymous variants can cause the premature termination of the codon (loss-of-function variants) or the substitution of a single base, leading to the formation of a modified protein (missense variants). DNA mutations can result from insertions, deletions or replacements of one or more nucleotides to a gene, as well as genetic alterations at the site of the boundary between an exon and an intron (splice site), thus affecting the RNA splicing and the structure of the final protein [21].

The interpretation of sequence variants is subject to rules and guidelines defined by the American College of Medical Genetics and Genomics in collaboration with the Association for Molecular Pathology, serving the international coordination and the commonly accepted terminology between different laboratories and research teams [22]. Thus, 5 types of variants are recognized according to their pathogenicity: (i) pathogenic, (ii) likely pathogenic, (iii) variants of uncertain significance, (iv) likely benign and (v) benign. The classification of each new mutation as causative for a disease should always be based on sufficient supporting evidence. Table 1 (Ref. [23, 24, 25, 26, 27, 28, 29, 30, 31, 32, 33, 34, 35, 36, 37, 38, 39, 40, 41, 42, 43, 44, 45, 46, 47, 48, 49, 50, 51, 52, 53, 54]) represents the most common DCM associated genes, the encoding protein and the expected clinical impact.

**Table 1.** **DCM associated genes, the resulting proteins and the phenotypic manifestations of disease-related variants**.

Genes	Encoded protein	Function	Frequency	Phenotypic features/Cardiac involvement
*Cytoskeleton- Z-disk genes*				
*DES* [23, 24, 25]	desmin	intermediated filaments with structural and mechanical properties	<1%	conduction system disease, elevated levels of creatinine kinase, heart failure development, LVEF reduction
*FLNC* [26, 27, 28]	filamin C	cross-liker between Z-disk and sarcolemma	∼3%	ECG low QRS voltages, LV fibrosis, overlap with ACM phenotype SCD, subepicardial scar on CMR, T-wave inversion
*NEXN* [29]	nexilin F-actin binding protein	adhesion and migration of cardiomyocytes	<1%	conduction defects, heart failure development, lethal fetal DCM with cardiomegaly and endocardial fibroelastosis
*VCL* [30]	vinculin	interacting protein that connects F-actin to the cell membrane and extracellular matrix	<1%	bradycardia, conduction defects, early onset DCM, overlap with HCM
*Desmosomal genes*				
*DSP* [28, 31, 32]	desmoplakin	component of desmosomes that maintain structural and communicative stability between cells	1–3%	subepicardial scar on CMR, LV impairment, LV fibrosis, PVCs, T wave inversions, episodic chest pain, troponin elevation
*Ion channel gene*				
*SCN5A* [33, 34]	sodium channel protein type 5 subunit α	a channel that controls sodium ions flow	<1%	long QT syndrome, conduction system disease, isolated ventricular dilation, hypokinesia
*Nuclear envelope gene*				
*LMNA* [35, 36]	lamin A/C	creates the lamina matrix at the nuclear envelope and participates in mitosis, nuclear stability and gene expression	∼4–7%	ventricular dilation, LV impairment, conduction system defects, syncope, ventricular arrhythmias, SCD
*Sarcomeric genes*				
*ACTC1* [37]	actin alpha cardiac muscle 1	located at the cytoplasm as part of the cytoskeleton	<1%	DCM phenotype, possible LV dilation, interstitial cardiac fibrosis
*ACTN2* [38, 39]	alpha actin 2	bundling protein that anchor actin and titin to Z-disks	<1%	ventricular arrhythmias, SCD, left ventricular non-compaction, LA and LV dilation, cardiac fibrosis
*MYH7* [40]	myosin-7	contributes to cardiac muscle contraction	∼3–5%	overlap with HCM, contractile disfunction, heart failure, atrial arrhythmias
*TNNC1* [41]	cardiac troponin C	allows the interaction of actin with myosin that result to the generation of tension, through the termination of the inhibitory action of cardiac troponin I	<1%	heart failure, sudden cardiac death, need for cardiac transplantation
*TNNI3* [42]	cardiac troponin I	blocks actin-myosin interaction thus facilitating muscle relaxation	<1%	heart failure, need for cardiac transplantation
*TNNT2* [41, 43]	cardiac troponin T	component of the sarcomere that modulates myocardial contraction	∼2%	overlap with HCM, early onset DCM, heart failure development, SCD
*TPMI* [44]	alpha-tropomyosin	involved in the cardiac muscle contraction through the regulation of the calcium-dependent interaction of actin and myosin	∼1–2%	heart failure, need for cardiac transplantation
*TTN* [45, 46, 47]	titin	connection between the Z-disk and the M-line thus facilitating contraction and relaxation of the cardiac muscle	∼15–25%	LV impairment, mid-wall fibrosis, sustained ventricular arrhythmias, heart failure
*Sarcoplasmic reticulum genes*				
*JPH2* [48]	junctophilin 2	membrane-binding protein that connects the plasma membrane with the sarcoplasmic reticulum	<1%	childhood-onset recessive DCM, association with HCM
*PLN* [49, 50]	phospholamban	located in sarcoplasmic reticulum and modulates contractility via calcium flow regulation	∼1%	conduction system defects, ventricular arrhythmias, cardiac fibrosis, heart failure, SCD
*Other genes*				
*BAG3* [51, 52]	BCL2-associated athanogene 3	co-chaperon for HSP70 and HSC70 chaperon proteins with anti-apoptotic properties	∼2%	conduction system defects, heart failure, early or late onset DCM, PVCs
*RBM20* [53, 54]	RNA-binding motif protein 20	splicing factor	∼2%	ventricular arrhythmias, SCD, LV impairment

ACM, arrhythmogenic cardiomyopathy; ARVC, arrhythmogenic right ventricular cardiomyopathy; CMR, cardiac magnetic resonance; DCM, dilated cardiomyopathy; ECG, electrocardiogram; HCM, hypertrophic cardiomyopathy; HSC70, heat shock cognate protein 70; HSP70, heat shock protein 70; LA, left atrium; LV, left ventricle; LVEF, left ventricular ejection fraction; PVCs, premature ventricular contractions; QRS, QRS complex; QT, QT interval; RBBB, right bundle branch block; RV, right ventricle; SCD, sudden cardiac death.

### 2.2 Genetic Substrate of DCM

Genetic causes associated with the development of dilated cardiomyopathy mainly involve mutations in genes encoding for proteins of the cytoskeleton, the cardiomyocytic sarcomere and the nuclear envelope, affecting the structural integrity of the myocyte and disturbing the normal function of the heart muscle [55]. Table 1 summarizes the DCM-related genes with evidence-based clinical validity according to the National Institute of Health (NIH) ClinGen classification, in comparison to their phenotypic features and cardiac involvement [56]. Subsequently, at the present section, 12 genes that have been identified as of “Definitive” and “Strong” evidence of disease association, will be described more specifically.

#### 2.2.1 Cytoskeletal Genes

Mutations in genes that encode proteins of the cytoskeleton could be a cause for DCM development. *FLNC* gene encodes filamin C, a protein that is mainly expressed in skeletal and cardiac muscles, permitting the grounding of the Z-disk to the intercalated domain. *FLNC* variants involve an increased risk for SCD since they are associated with ventricular arrhythmias induction and cardiac arrest in relatively young individuals [27, 57, 58]. Consequently, the threshold for primary ICD implantation in this group of patients is considered lower, compared to the general DCM population [14].

Desmin is an intermediate filament protein encoded by the *DES* gene that is associated with both skeletal and cardiac myopathies [24]. Variants in this gene have been described as causative for SCD and arrhythmias manifestation in the affected individuals [59].

#### 2.2.2 Desmosomal Gene

Desmosomes are intercellular structures that connect neighboring cells, thus betraying mechanical strength to cardiac tissue [60]. Mutations in genes that encode desmosomal proteins can disturb the normal function of the left ventricle causing both arrhythmogenic and dilated cardiomyopathy [31]. Pathogenic variants in *DSP *gene that modify the basic function of desmoplakin protein are associated with sustained ventricular arrhythmias and cardiac fibrosis in patients with DCM. As a result, their detection in individuals and families demands a careful evaluation and more aggressive preventive management due to the increased risk of SCD [61].

#### 2.2.3 Ion Channel Gene

*SCN5A* gene encodes the sodium channel protein and is involved in many different cardiac syndromic conditions such as Brugada syndrome, long-QT syndrome, sudden infant death syndrome as well as, conduction disorders. Evidence based data suggest that mutations in this gene may contribute to DCM development with arrhythmic phenotypic manifestations including frequent premature ventricular complexes, atrioventricular block and SCD [34, 57].

#### 2.2.4 Nuclear Envelope Gene

*LMNA* gene encodes the proteins Lamin A and C which are dominant components of the nuclear envelope. Except from their structural role, lamin family proteins are considered valuable regulators of gene expression affecting chromatin organization, genome replication and the integrity of multiple molecular pathways [35]. *LMNA* mutations result to a spectrum of heterogeneous syndromic diseases called laminopathies, such as muscular dystrophies (Emery-Dreifuss, Limb Girdle), lipodystrophies (Dunnigan-type familial partial lipodystrophy) and progeria syndromes [36]. DCM could also result from an inherited cause associated with the *LMNA *gene.

*LMNA* mutations are detected approximately to 1 out of 10 patients with genetic DCM. Although the incidence is much lower in comparison to the *TTNtvs*, these mutations involve a significant clinical impact associated with arrhythmogenicity and disorders of the cardiac conduction system [62]. Specifically, it has been reported that DCM patients carrying a pathogenic or likely pathogenic LMNA variant are at risk for the development of malignant ventricular arrhythmias and SCD, even if a LVEF more than 35% is preserved [15]. Consequently, in 2015 the European guidelines included to their recommendations the thorough evaluation of patients with confirmed LMNA-associated DCM as they could be candidates to receive an implantable cardiac defibrillator as part of primary SCD prevention therapy [12].

#### 2.2.5 Sarcomeric Genes

Sarcomeric genes such as *TNNT2*, *TNNC1* and *MYH7* have been demonstrated as DCM related, since mutations in these genes contribute also to the development of progressive heart failure and cardiac transplantation, as well as atrial arrhythmias [40, 41]. However, mutations in genes that encode proteins of the troponin complex demonstrate 100% penetrance and lower mean age of diagnosis in contrast to mutations of the *MYH7* gene that are associated with late onset of the disease and incomplete penetrance [63].

In addition, *TTN* gene encodes titin protein, a dominant element of the sarcomere, which is the myocyte unit responsible for the cardiac muscle contraction and relaxation. More precisely, titin is the largest protein of the human body and the connecting link between the Z-disk and the thick filaments of the M-line, providing structural stability, expansibility, signal sensing and transduction to the sarcomere [47]. Titin truncating variants (*TTNtv*) are the most common variants detected in DCM, accounting for approximately 15–25% of the cases. They are also detected in the 2% of individuals without diagnosed cardiomyopathy. Therefore, different variants of this gene involve different clinical impact thus making the assessment of a titin polymorphism a challenging process, including both the risk to underestimate and to overestimate its pathogenicity. In order to increase the accuracy of pathogenic *TTNtv* identification, a calculating tool termed PSI has been proposed. It is based on a scoring system that measures the proportion of *TTN* transcripts that include a given exon and expressed in human cardiac tissue. A high PSI score indicate an increased probability of pathogenicity [45, 47].

*TTNtvs* are frequently associated with a mild form of DCM that often results to a reverse remodeling of the left ventricle, if a patient follows optimal medical treatment [21, 57]. In comparison to other DCM causes, such as *LMNA* mutations or idiopathic DCM, patients with detected *TTNtv*are less prone to significant reduction of LVEF (≤35%), while usually express the disease at a higher age and present more prolonged life expectancy [64]. However, *TTN *gene variants remain an important risk factor associated with arrhythmia manifestation and adverse outcome in DCM patients, especially when coexists with fibrotic heart lesions, detected on cardiac magnetic resonance (CMR) [46].

#### 2.2.6 Sarcoplasmic Reticulum Gene

Phospholamban is a transmembrane protein encoded by the *PLN* gene. This protein is located at the sarcoplasmic reticulum (SR) and regulates the function of the SR ${\mathrm{Ca}}^{2+}$adenosin triphosphatase isoform 2a (SERCA2a) pump, depending on its phosphorylation phase. SERCA2a is an intracellular protein responsible for the entry of calcium into the SR, leading to the reduction of its cytosolic concentration, thus triggering cardiac relaxation and influencing contractility [65].

The frequency of *PLN *mutations in DCM patients is approximately 2% with a slightly higher proportion of female carriers [66]. Only a few mutations of this gene have been recorded as DCM causative. They include both loss-of-function variants that inhibit completely or partially the interaction with SERCA2a pump, or missense variants that modify the normal function and phosphorylation of the protein, leading to abnormal calcium handling and resulting to altered contractile properties of cardiomyocytes [49]. In DCM patients, PLN mutations present an autonomic recessive inheritance and are associated with early onset severe heart failure development and high frequency of ventricular arrhythmias [50, 66].

#### 2.2.7 Other Genes

*BAG3* and *RBM20 *genes encode proteins responsible for apoptosis inhibition and splicing regulation of cardiac DNA, respectively. Mutations in both genes result to a severe form of DCM including high rate of SCD or need for cardiac transplantation in a young age, thus demanding accurate risk assessment and appropriate treatment [51, 54].

## 3. Precision Medicine in DCM Patients

**Fig. 1.**
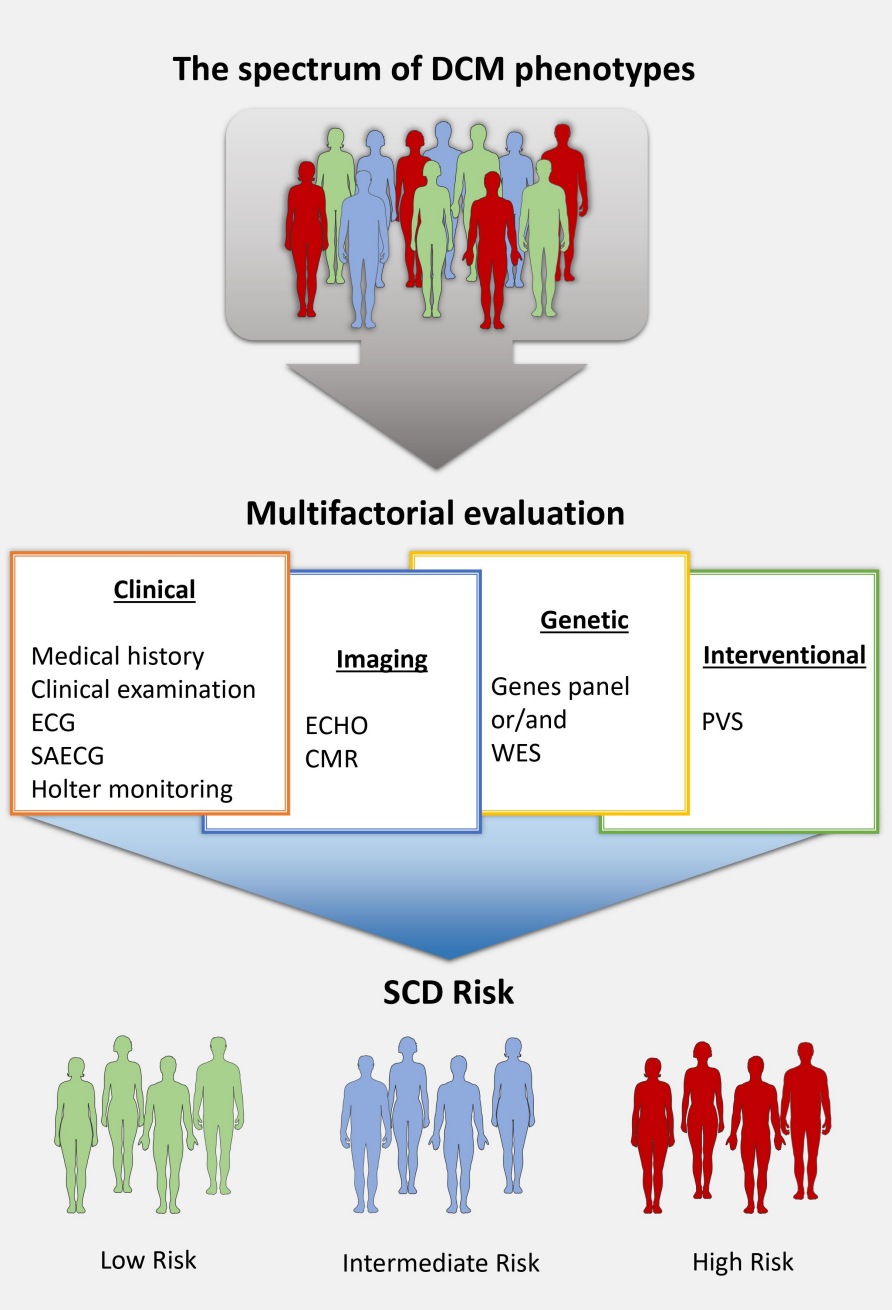
**The optimal multifactorial assessment of DCM patients**. The SCD risk stratification into three groups (low, intermediate and high risk) is based on the protocol of the ReCONSIDER trial (NCT04246450) [12]. CMR, cardiac magnetic resonance; DCM, dilated cardiomyopathy; ECG, electrocardiogram; ECHO, echocardiogram; PVS, programmed ventricular stimulation; SAECG, signal-average electrocardiogram; WES, whole exome sequencing.

The first step for the optimal management of DCM is the identification of patients at high risk, who will benefit the most from ICD implantation. The diversity of the disease imposes the concurrent evaluation of multiple risk factors including genetics, environmental and clinical, in order to achieve a greater accuracy regarding diagnosis and therapeutic decision making. The idea of a straight correlation between genotype and phenotype is not considered scientifically acceptable as it is known that many other factors, like gender, age, comorbidities and lifestyle, usually affect both the expressivity and penetrance of a mutated gene (Fig. 1, Ref. [12]) [67].

### The Value of Risk Factors beyond LVEF

LVEF is a dominant parameter of the assessment of DCM patients. In combination with the New York Heart Association (NYHA) classification of heart failure functional status, they compose the selection criteria for the detection of high-risk patients who should receive a defibrillator device [14]. However, previous clinical studies could not demonstrate an overall survival benefit after ICD preventive treatment in DCM patients who evaluated based on LVEF [68, 69]. The DANISH trial followed a cohort of 556 individuals with non-ischemic heart failure for a median period of 5.5 years, resulting that the prophylactic ICD therapy was not associated with a decreased rate of death from any cause [70]. This evidence suggests that the selection process of high-risk patients’ needs improvement by evaluating further factors with validated predictive value [71].

Programmed ventricular stimulation (PVS) could play an important role in SCD prevention [72]. A study by Gatzoulis *et al*. [73] examined 158 patients with idiopathic DCM who submitted to PVS. The final decision for ICD implantation was received according to the outcome of PVS in combination with the current factors of LVEF and NYHA classification. Among the main findings of the study the authors demonstrated that the provocation of ventricular tachyarrhythmias during PVS is the only independent factor that predicts the long-term activation of an ICD. Other factors that also seem to provide a subsequent predictive value by detecting patients with impaired cardiac function, at risk for arrhythmias development and mortality, are the deceleration capacity of heart rate, the late potentials recorded from the signal average electrocardiogram, the T wave alternans interpreted from holter monitoring and the late gadolinium enhancement on CMR, reflecting the areas of regional fibrosis and permitting the assessment of scar tissue in myocardium [74, 75, 76, 77, 78].

## 4. The Role of Genetic Evaluation in Patients’ Management

### 4.1 Gene-Based Personalized Approach in DCM Patients: Pros

A personalized approach should result to the selection of the optimal treatment, for the right patient at the appropriate time. Genetic testing could definitely play an important role as a dominant part of this method, providing useful information for the initial causes of the disease, thus contributing to the most essential understanding of the causative background [67]. Moreover, genetic counseling can inform both the patients and its family about the potential benefits and drawbacks as well as the availability of the suggested treatments [79]. Family screening could also detect asymptomatic carriers of pathogenic mutations thus allowing the timely intervention and/or the regular follow up [5]. The detection of the affected population at an earlier disease stage could result to survival improvement after the administration of the suitable medical and interventional treatment [80].

### 4.2 Gene-Based Personalized Approach in DCM Patients: Cons

However, genetic testing comprehends significant caveats that shouldn’t be ignored during the evaluation of patients with DCM. The interpretation of genetic results is still under investigation as a large number of detected variants do not have a proven clinical impact (variants of unknown significance), thus confusing the physicians about the appropriate treatment and clinical council of the patients [81]. Furthermore, even if a pathogenic or likely pathogenic mutation will be detected the possibility of a proband to develop the disease phenotype as well as, the time and severity of the clinical manifestations remains undetermined for the majority of cases [11]. According to the latest guidelines, 5 genes (*LMNA*, *RBM20*, *PLN*, *FLN *and *TTN*) are considered as of higher risk for the development of SCD and atrial or ventricular tachyarrhythmias. The detection of pathogenic or likely pathogenic variants in these genes should raise suspicions regarding the onset of adverse cardiac events. As a result, the early indication for primary prevention with an ICD implantation, is suggested [14]. This recommendation raises concerns and clinical dilemmas when refers to young patients under 40 years of age without comorbidities or aggressive phenotypic expression of the disease, especially when the benefit is weighed against the cost of a long-term implantable device.

## 5. Discussion

The beneficial role of genetic testing in hereditary cardiomyopathies and more especially in DCM, is currently generally accepted, since the valuable contribution of genetic evaluation in diagnosis, prognosis, family screening and reproductive planning has been demonstrated through multiple studies and case reports [82]. Furthermore, risk stratification based on the reduced LVEF can recognize patients at high risk for SCD, however, its sensitivity and specificity are low [83]. As a result, the significant role of a multifactorial model for patients’ assessment and the essential involvement of genome, have been proposed as key elements for optimal clinical management [14, 83].

In the future, the improvement of genetic testing regarding the selection of genes panel and the accurate interpretation of the subsequent results will enhance its contribution in daily clinical practice [82]. Moreover, the implementation of gene targeted therapies, both in terms of prevention and symptomatic patients’ relief based on pathogenic gene mutations, and in terms of directed therapies that turn against DCM-causative molecular mechanisms are intensively explored. In combination, the novel technology of next-generation sequencing for human genome is becoming more affordable and widely applied [84].

Currently, there are several aspects regarding the role of genetic testing in DCM risk stratification that have not been fully elucidated. There are many genes with limited evidence of their clinical validity and variants of uncertain significance that indicate the need for further research [56, 85]. The contribution of comprehensive genetic testing for cardiomyopathies and arrhythmias is still under investigation while newer data show its potential value in the diagnosis and treatment of patients without phenotypic manifestation of the disease [86]. In addition, large studies that demonstrate the association between genotype and SCD risk in DCM patients are still missing, while, the wide and diverse spectrum of genotype-phenotype correlation, complicates the prediction of clinical impact and disease severity [83]. Finally, in order to achieve optimal patients’ diagnosis and management a team approach is required that will include experts of different medical fields, associated with hereditary cardiomyopathies, such as cardiologists, genetic counsellors and/or medical genetics [82].

## 6. Conclusions

DCM is a cardiac disorder with a diverse genetic architecture, which is not yet completely understood regarding its involvement in the onset and development of the disease, as well as in the manifestation of the different phenotypes. The current management of DCM patients should be modified as newer data regarding the causative mechanisms are gradually better understood. Determining the direct correlation between the genotype and the phenotype of this disease will allow the transition from genetic research to the implementation of individualized therapies that reduce the risk of sudden cardiac death and improve patients’ quality of life.

## Abbreviations

ACM, arrhythmogenic cardiomyopathy; ARVC, arrhythmogenic right ventricular cardiomyopathy; CMR, cardiac magnetic resonance; DCM, dilated cardiomyopathy; DNA, deoxyribonucleic acid; ECG, electrocardiogram; ECHO, echocardiogram; HCM, hypertrophic cardiomyopathy; HSC70, heat shock cognate protein 70; HSP70, heat shock protein 70; LA, left atrium; LMNA, lamin A/C; LV, left ventricle; LVEF, left ventricular ejection fraction; PVS, programmed ventricular stimulation; QRS, QRS complex; QT, QT interval; RBBB, right bundle branch block; RV, right ventricle; RNA, ribonucleic acid; SCD, sudden cardiac death; SNP, single-nucleotide polymorphism; SR, sarcoplasmic reticulum; SAECG, signal-average electrocardiogram; SERCA2a, sarcoplasmic reticulum ${\mathrm{Ca}}^{2+}$-adenosin triphosphatase isoform 2a; WES, whole exome sequencing.
